# Identification of extended-spectrum beta-lactamase (CTX-M)-producing *Klebsiella pneumoniae* belonging to ST37, ST290, and ST2640 in captive giant pandas

**DOI:** 10.1186/s12917-022-03276-7

**Published:** 2022-05-17

**Authors:** Xiaoyan Su, Xia Yan, Yunli Li, Dongsheng Zhang, Lin Li, Yi Geng, Fei Su, Chanjuan Yue, Rong Hou, Songrui Liu

**Affiliations:** 1 Sichuan Key Laboratory of Conservation Biology for Endangered Wildlife, Chengdu Research Base of Giant Panda Breeding, Chengdu, 610081 China; 2 College of Veterinary Medicine, Sichuan Agricultural University, Chengdu, 611130 China; 3 Institute of Animal Husbandry and Veterinary Science, Zhejiang Academy of Agricultural Sciences, Hangzhou, 310021 China

**Keywords:** ESBL-producing *Klebsiella pneumoniae*, Antimicrobial resistance, Epidemiology, Giant panda

## Abstract

**Background:**

Extended-spectrum β-lactamases (ESBL)-producing strains of *Klebsiella pneumoniae* remain a worldwide, critical clinical concern. However, limited information was available concerning ESBL-producing *Klebsiella pneumoniae* in giant pandas. The objective of this study was to characterize ESBL-producing *Klebsiella pneumoniae* isolates from captive giant pandas. A total of 211 *Klebsiella pneumoniae* isolates were collected from 108 giant pandas housed at the Chengdu Research Base of Giant Panda Breeding (CRBGP), China. Samples were screened for the ESBL-producing phenotype via the double-disk synergy test.

**Result:**

A total of three (1.42%, *n* = 3/211) ESBL-producing *Klebsiella pneumoniae* strains were identified, and characterization of ESBL-producing *Klebsiella pneumoniae* isolates were studied by the detection of ESBL genes and mobile genetic elements (MGEs), evaluation of antimicrobial susceptibility and detection of associated resistance genes. Clonal analysis was performed by multi-locus sequencing type (MLST). Among the three ESBL-producing isolates, different ESBL-encoding genes, including *bla*_CTX-M_, and *bla*_TEM,_ were detected. These three isolates were found to carry MGEs genes (i.e., IS903 and *tnpU*) and antimicrobial resistance genes (i.e., *aac(6')-Ib*, *aac(6')-I*, *qnrA*, and *qnrB*). Furthermore, it was found that the three isolates were not hypermucoviscosity, resistant to at least 13 antibiotics and belonged to different ST types (ST37, ST290, and ST2640).

**Conclusion:**

Effective surveillance and strict infection control strategies should be implemented to prevent outbreaks of ESBL-producing *Klebsiella pneumoniae* in giant pandas.

## Background

*Klebsiella pneumoniae (K. pneumoniae)* is an important human pathogen causing numerous infections in hospitals, long-term care facilities, and are associated with community-acquired infection. The bacteria infect the lungs, urinary tract, and surgical sites, causing soft tissue infections and bacteremia [1]. The giant panda, *Ailuropoda melanoleuca*, is one of the world’s most recognized conservation dependent species and is only distributed in the mountainous areas of Sichuan, Gansu, and Shaanxi provinces [2]. The results of the fourth census of wild giant pandas showed that the population has reached 1864 [3], and the current population of captive giant pandas reached 600. With the growth of the captive population of giant pandas and the in-depth study of their diseases, infections with *K. pneumoniae* have emerged. Wang *et. al* (1998) reported that giant pandas infected with *K. pneumoniae* developed hemorrhagic enteritis [4]. Later, a sub-adult giant panda was diagnosed with *K. pneumoniae* and *Escherichia coli* infection and subsequently died from hemorrhagic sepsis, with the isolated *K. pneumoniae* found to be pathogenic to mice [5]. Subsequent cases of genital hematuria, enteritis, and sepsis caused by *K. pneumoniae* infection in giant pandas were reported [6]. We conducted an etiological study on a dead giant panda found in a nature reserve in Sichuan and discovered the giant panda died of multiple organ dysfunction syndrome caused by *K. pneumoniae* and *Proteus mirabilis* infection [7]. This showed that *K. pneumoniae* infection was a serious threat to the life and health of giant pandas. In addition, studies had shown that *K. pneumoniae* can be transmitted through the air [8]; therefore, captive animals infected with *K. pneumoniae* may be a source of zoonotic infection for husbandry staff and even visitors to wildlife centers.

The emergence of antibiotic resistance is an increasingly alarming public health threat due to the fact they undermine the efficacy of antibiotic treatment [9]. Furthermore, it is expected that antibiotic resistance will be the leading cause of global mortality by 2050, possibly exceeding that of cancer [10]. β-lactam antibiotics are among the most frequently prescribed antimicrobials. However, extended-spectrum β-lactamases (ESBL) have emerged in numerous hospitals worldwide. These enzymes confered resistance to extended-spectrum cephalosporins and related oxyimino-β-lactams (ceftazidime, cefotaxime, and aztreonam) but were predominantly sensitive to carbapenems, cephamycins, and β-lactamase inhibitors such as clavulanic acid [11]. Antibiotics containing β-lactams are commonly used in disease prevention and control in captive pandas. Antibiotics used in giant pandas indicated that β-lactam antibiotics account for about 50% of total usage, while carbapenem antibiotics including imipenem also were used in Chengdu Research Base of Giant Panda Breeding (CRBGP), China. In the field of human and livestock medicine, researchers have conducted in-depth studies on the clinical isolation of ESBL-producing *K. pneumoniae* regarding drug resistance, genetic toxicity, and molecular epidemiology, and these results have played a positive role in the scientific and effective prevention and control of ESBL-producing *K. pneumoniae* [12–15]. However, molecular epidemiology studies of ESBL-producing *K. pneumoniae* in giant pandas are currently lacking. Therefore, this study aimed to isolate ESBL-producing *K. pneumoniae* from captive giant pandas, explore the prevalence and genotype of ESBL-producing *K. pneumoniae*, provide a scientific basis for the clinical use of antibiotics, and provide systematic experimental data and a reference basis for preventing and controlling the spread of such bacteria.

## Results

### Prevalence of ESBL-producing isolates and ESBL-encoding genes

**Fig. 1:**
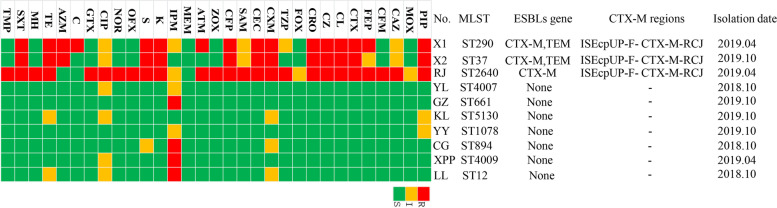
The identified antimicrobial susceptibility profiles of *K. pneumoniae* isolates and ESBL genotype. X1, X2, and RJ are ESBL- positive *K. pneumoniae* isolates. YL, GZ, KL, YY, CG, XPP, and LL are non- ESBL positive *K. pneumoniae* isolates as controls. PIP, Piperacillin; MOX, Moxalactam; CAZ, Ceftazidime; CFM, Cefixime; FEP, Cefepime; CTX, Cefotaxime; CL, Cephalexin; CZ, Caphazolin; CRO, Ceftriaxone; FOX, Cefoxitin; TZP, Piperacillin/Tazobactam; CXM, Cefuroxime; CEC, Cefaclor; SAM, Ampicillin/Sulbactam; CFP, Cefoperazone; ZOX, Ceftizoxime; ATM, Aztreonam; MEM, Meropenem; IPM, Imipenem; K, Kanamycin; S, Streptomycin; OFX, Ofloxacin; NOR, Norfloxacin; CIP, Ciprofloxacin; GTX, Gatifloxacin; C, Chloramphenicol; AZM, Azithromycin; TE, Doxycycline; MH, Minocycline; SXT, Compound Sulfamethoxazole; TMP, trimethoprim. "None" indicates a negative detection,“-” indicates no test. R, resistance; I, intermediary sensitive; S, sensitive

Three ESBL-producing isolates were detected with a prevalence of 1.42% (*n* = 3/211) in the 108 giant pandas listed. Five ESBL-encoding genes were identified including *bla*_TEM_ (*n* = 2), and *bla*_CTX-M_ (*n* = 3). Two isolates were detected co-carrying *bla*_TEM_, *bla*_CTX-M_, and one carrying *bla*_CTX-M_ (Fig. 1).

### String test

The string test results showed that the mucoid string of three ESBL-producing *K. pneumonia* strains was all lower than 5 mm, indicating that the string test of the three strains were all negative, and further defined that the three strains were not hypermucoviscous.

### Antimicrobial resistance profiles

The three ESBL-producing isolates were all (*n* = 3, 100%) resistant to piperacillin, cefotaxime, cephalexin, caphazolin, ceftriaxone, cefuroxime, cefaclor, cefoperazone, kanamycin, streptomycin, doxycycline, and compound sulfamethoxazole (Fig. 1). Isolate X1 was resistant to 16 antibiotics. Isolate X2 was resistant to 13 antibiotics, while isolate RJ was resistant to 25 antibiotics; however, seven non-ESBL-producing isolates were still high resistant to imipenem (57.14%).

### Molecular characteristics of ESBL-producing isolates

**Fig. 2:**
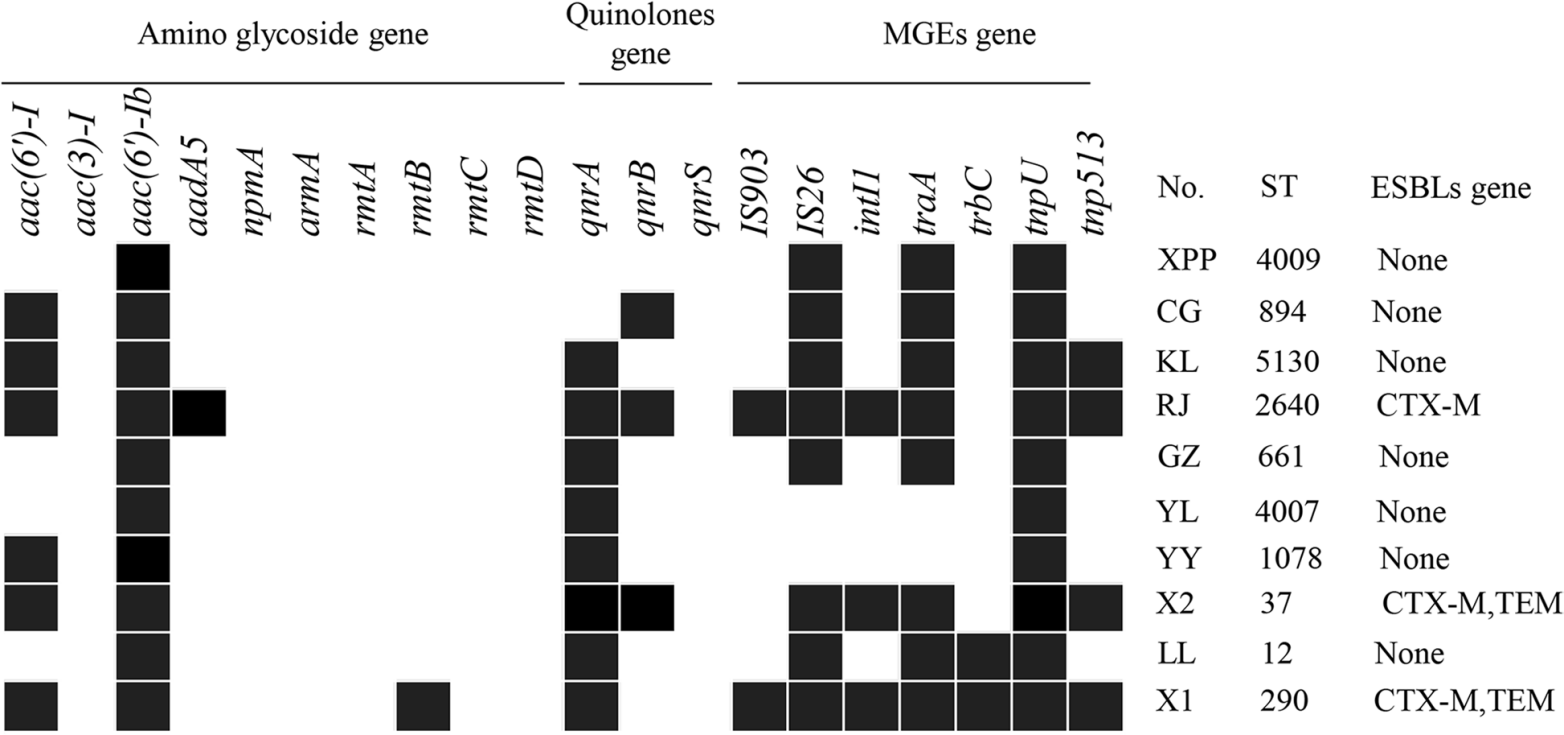
Genetic features of 10 *K. pneumoniae* isolated from giant pandas recovered in 2018 and 2019 with the respective ESBL genes, MGEs gene, and antimicrobial resistance genes. X1, X2, and RJ are ESBL- positive *K. pneumoniae* isolates. YL, GZ, KL, YY, CG, XPP, and LL are non- ESBL positive *K. pneumoniae* isolates as controls

ESBL-producing isolates X2 and X1 were detected co-carrying two ESBL encoding genes (*bla*_CTX-M_ and *bla*_TEM_), while RJ carried one ESBL-encoding genes (*bla*_CTX-M_). Among the three ESBL isolates, three STs, ST2640, ST290, and ST37, were identified (Fig. 2).

### Distribution of MGEs genes and antimicrobial resistance genes

All three ESBL-producing isolates and seven non-ESBL-producing isolates tested were carrying at least one of the seven investigated MGEs genes and one of thirteen antimicrobial resistance genes. Three ESBL-producing isolates were carrying 4–5 antimicrobial resistance genes and about 6–7 MGEs genes. Seven non-ESBL-producing isolates were carrying about 1–3 antimicrobial resistance genes and 1–4 MGEs genes. All isolates were carrying *aac(6')-Ib* and *tnpU*. Compared with the seven non-ESBL-producing isolates, the three ESBL-producing isolates carried *aadA5*, *rmtB*, *IS903*, and *intI1* (Fig. 2). The PCR results showed that *ISEcpUP-F- CTX-M-RCJ* was positive detected, which means the *bla*_CTX-M_ was carried by class 1 integron (Fig. 1).

### MLST characteristics of ESBL-producing isolates

**Fig. 3:**
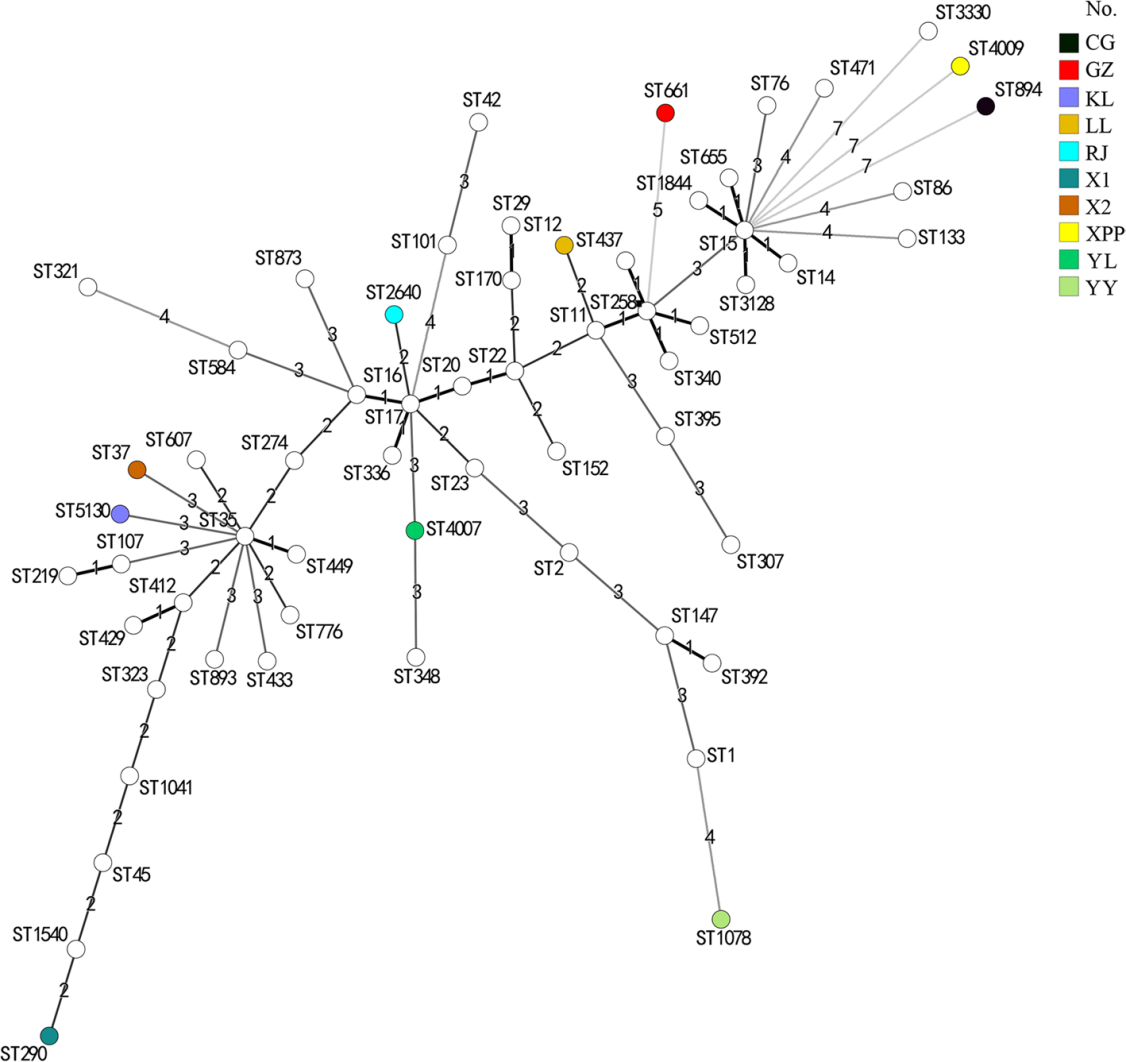
The minimum spanning tree of *K. pneumoniae* isolated from giant pandas. The MLST analysis were based on the ST types of 3 ESBL-positive *K. pneumoniae* (X1, X2, RJ), 7 non-ESBL-positive *K. pneumoniae* (YL, GZ, KL, YY, CG, XPP), isolated from giant pandas and 51 other common ESBL-production *K. pneumoniae* ST types using BioNumerics version 7.6 software. Colour coding corresponds to 10 *K. pneumoniae* isolated from giant pandas. The number on branches correspond the different loci batwing between different ST-types

The MLST is a nucleotide sequence-based method that is adequate for characterizing the genetic relationships among bacterial isolates. The result showed that the distribution of ST type of *K. pneumoniae* from giant panda was dispersed and presented diversity. The 3 ESBL-positive *K. pneumoniae* were on different branches, which indicated they had a distant relationship, and at least two allele variants in the ST type existed in the common ESBLs-producing *K. pneumoniae* (Fig. 3).

## Discussion

ESBL-producing *K. pneumoniae* have been frequently encountered worldwide, especially in hospitals. However, ESBL-producing *K. pneumoniae* are rarely found in wild animals such as the giant panda [16–18]. Therefore, this study intended to analyze the prevalence, genotype, and antimicrobial susceptibility profiles of ESBL-producing *K. pneumoniae* collected from captive giant pandas. The result showed that the prevalence of ESBL-producing *strains* among *K. pneumoniae* isolates from captive giant pandas at the CRBGP was 1.42% (*n* = 3/211). The prevalence of this ESBL-producing *K. pneumoniae* in giant pandas was lower than in livestock animals and livestock animal products. In the north-west province of South Africa, the most common ESBL-producing bacteria from cattle farms and raw beef was *E. coli* (58.2%), followed by *K. pneumoniae* (41.8%) [18]. ESBL-s producing *K. pneumoniae* collected from adult cattle was 23.4% in North Lebanon [19]. These ESBL-producing *K. pneumoniae* isolates were reported as 53% from diseased dogs in a Veterinary Teaching Hospital in Beijing, China [17], 8.75% were found from raw bulk tank milk of dairy farms in Indonesia [16], 10.9% were detected in cow milk in eastern and north-western India [20] and 17.5% were from companion animals in Europe [21]. The prevalence of ESBL-producing *K. pneumoniae* in giant pandas was lower than in humans also. The most common ESBL-producing Enterobacteriaceae detected in a children’s hospital in Japan was *E. coli* (79.8%), followed by *K. pneumoniae* (9.1%) [22]. In Iran, the prevalence of ESBL-producing *K. pneumoniae* was 43.5% (95% CI 39.3–47.9%) among clinical *K. pneumoniae* isolates [23]. In Spain, the prevalence of these bacteria in 2017 was 7.2% [24]. In a similar study conducted in Canada from 2010–2012, Karlowsky et al. [25] reported that the prevalence of ESBL-producers was 3.6% among *K. pneumoniae* isolates [25]. Studies in Saudi Arabia, United Arab Emirates, and Tunisia between 2009 and 2018 revealed a prevalence of these pathogenic bacteria between 38 and 55% in different community settings and hospitals [23]. The prevalence of this pathogenic bacteria was 59.2% among clinical *K. pneumoniae* isolates in Iran [26]. A possible explanation for the lower prevalence of ESBL-producing *K. pneumoniae* in giant pandas is the less frequent use of antibiotics in giant pandas than in humans and domestic animals.

In this study, three ESBL-producing *K. pneumoniae* isolates were detected co-carrying 1 ~ 2 ESBL-encoding genes, including *bla*_TEM_ and *bla*_CTX-M_. Zhou et al. also reported that ESBL-producing *E. coli* carrying CTX-M gene were detected in captive giant panda in Shanghai, China. Therefore, *bla*_*CTX-M*_ may be the main prevalent ESBL-encoding genes in captive giant pandas [27]. Our result was similar to that described in previous studies, where TEM- and CTX-M- types were the main families of ESBL [11, 28]. Furthermore, reports have indicated that CTX-Ms have been rapidly disseminated among populations of gram-negative bacteria in clinical settings in recent years [29, 30]. The blaCTX-M was always connected with mobile genetic elements (MGEs) such as integrons, transposons, plasmid and it caused the horizontal gene spread between the same or different species of bacteria [31].

While partial MGEs were detected in this study, including eight different types of MGEs genes, non-ESBL-producing *K. pneumoniae* strains carried significantly fewer MGEs genes (*n* = 2 ~ 3), and the results indicated that the *bla*_CTX-M_ was carried by the class 1 integron. Based on the fact that ESBL-producing *K. pneumoniae* was rarely found in captive giant pandas, we suspected that these resistance genes were transmitted by MGEs, and may have been transmitted from humans or other local animals to the captive giant pandas.

The three ESBL-producing *K. pneumoniae* isolates showed high resistance to many antimicrobial agents (more than 13 antibiotics). In our study, Meropenem was effective against the three ESBL-producing *K. pneumoniae* isolates, and IPM was effective against about 10% of the strains (*n* = 10). The three ESBL-producing *K. pneumoniae* isolates were carrying 5 antimicrobial resistance genes, *aac(6')-Ib*, *tnpU*, *aac(6')-I*, *qnrA*, and *qnrB*, which are associated with aminoglycoside and quinolones resistance. In China, ESBL-positive *K. pneumoniae* strains were > 70% susceptible only to IPM, ertapenem, or amikacin [32]. Similar studies have reported that although the majority of ESBL-producing strains could tolerate high concentrations of most cephalosporins and fluoroquinolones, none exhibited resistance to carbapenems (i.e., imipenem and meropenem) [33]. Interestingly, some of these non-ESBL-producing *K. pneumoniae* strains were detected carrying aminoglycoside and quinolones-encoding genes that are associated with aminoglycoside and quinolones resistance, however, none of them exhibited resistance to those antimicrobial agents. The reason may be partly due to the expression levels of aminoglycoside and quinolones-encoding genes being quite low in most cases [34].

Previous studies have reported that about 50% of the antibiotic prescriptions for the treatment of *K. pneumonia* infection were inappropriate and may cause the emergence of antimicrobial resistance (AMR) [35]. We recommend that veterinary professionals paied close attention to the choice of antimicrobial agents to prevent widespread AMR emergence in captive giant pandas.

ST37 was simultaneously shown to be closely associated with ESBLs [36]. And it was thought to be shared between humans and animals [37]. MLST revealed that the three ESBL-positive isolates from giant pandas were ST37, ST290, and ST2640, which means they have a distant relationship. However, ST37 ESBL-positive isolates in pandas were consistent with the main epidemic ST types in China. ST290 was reported to be related with infection of humans and cattle in China, the United States, and Australia [14]. Furthermore, hypervirulent strains of ST290 were detected in healthy captive red kangaroo in Zhengzhou Zoo, China, and also healthy pigs and humans in Thailand [14, 38]. ST2640 *K. pneumoniae* was detected in bloodstream infection of human in Taiwan [39]. Although ST290 and ST2640 is not a common type of bacteria in China, it was still found in giant pandas.

In conclusion, this was the first ever attempt to study the occurrence and characterization of ESBL-producing *K. pneumoniae* in giant pandas. The result showed that the ESBL-producing *K. pneumoniae* had existed in giant pandas. But the route of bacterial transmission was not clear. It could be that the use of antibiotics caused genetic mutations in the bacteria, or the horizontal transmission between bacterial, or the transmission by humans or animals in giant panda habitats. Some new techniques that prevented bacterial antibiotic resistance were reported. Mode-of-action-guided chemical modifications of compounds and the development of new antibiotics was considered desirable in dealing with bacterial antibiotic resistance [40]. The successful use of pneumococcal vaccine has brought hope to the use of vaccine against bacterial diseases [41]. Chinese medicine has also been actively applied to bacterial infection [42]. These successful studies may be desirable measures for the prevention and treatment of bacterial diseases in giant pandas in the future, but more research is needed.

Based on the high-frequency resistant to antibiotic of this pathogen, we recommend that the enhancing monitor of bacterial resistance of giant pandas should be performed in clinical veterinarians to facilitate the rational use of antibiotics. And the environmental disinfection of giant panda habitat is also necessary. That may prevent the widespread of ESBL-producing *K. pneumoniae* emergence in the giant pandas.

### Limitations of the study

We studied antimicrobial resistance genes and antimicrobial resistance phenotypes of ESBL-producing *K. pneumoniae*, but it is not clear how these antimicrobial resistance genes regulated the drug resistance phenotypes, and the transmission mechanism of these antimicrobial resistance genes. In a future study, we will further investigate whether antimicrobial resistance genes locate in plasmids and their horizontal transmission mechanisms.

## Methods

### Subjects

#### Bacterial isolates and screening for ESBL phenotype

Two hundred and eleven nonduplicated *K. pneumoniae* isolates were collected from 376 fresh feces of 108 captive giant pandas (female: *n* = 54, male: *n* = 54, age: 5–22 years) housed at CRBGP in Sichuan, China during 2018 to 2019. These isolates were identified as *K. pneumoniae* by Gram staining, 16 s rDNA, and bacterial biochemical identification. All of the isolates were tested for ESBL production by the CLSI-recommended confirmatory double-disc combination test (CLSI, 2019). All of the isolates were screened for ESBL production using cefotaxime (CTX) and ceftazidime (CAZ) alone and in combination with clavulanic acid according to the double-disk synergy test method (DDST) (CLSI, 2019). Phenotypic presence of ESBL in the isolates was determined by detecting diameter enhancement of the inhibition zone of the clavulanate disk and corresponding β-lactam antimicrobial disk. If the enhancement value was > 5 mm, the isolate was presumed to be an ESBL producer [38].

### String test

Isolates were cultured on blood agar plates incubated overnight at 37 °C. An inoculating loop was used to touch the colonies gently and lift. Hypermucoviscosity (HV) was defined as a mucoid string > 5 mm in length observed visually (positive string test).

### Antimicrobial susceptibility testing

Antimicrobial susceptibility tests of the three ESBL-producing isolates and seven non-ESBL-producing isolates were evaluated using the disk diffusion method to Piperacillin 100 μg (PIP), Moxalactam 30 μg (MOX), Ceftazidime 30 μg (CAZ), Cefixime 5 μg (CFM), Cefepime 30 μg (FEP), Cefotaxime 30 μg (CTX), Cephalexin 30 μg (CL), Caphazolin 30 μg (CZ), Ceftriaxone 30 μg (CRO), Cefoxitin 30 μg (FOX), Piperacillin/Tazobactam 100/10 μg (TZP), Cefuroxime 30 μg (CXM), Cefaclor 30 μg (CEC), Ampicillin/Sulbactam 10/10 μg (SAM), Cefoperazone 75 μg (CFP), Ceftizoxime 30 μg (ZOX), Aztreonam 30 μg (ATM), Meropenem 10 μg (MEM), Imipenem 10 μg (IPM), Kanamycin 30 μg (K), Streptomycin 10 μg (S), Ofloxacin 5 μg (OFX), Norfloxacin 10 μg (NOR), Ciprofloxacin 5 μg (CIP); Gatifloxacin 5 μg (GTX), Chloramphenicol 30 μg (C), Azithromycin 15 μg (AZM), Doxycycline 30 μg (TE), Minocycline 30 μg (MH), Compound Sulfamethoxazole 23.75/1.25 μg (SMZ), and Trimethoprim 5 μg (TMP) (Oxoid, Basingstoke, United Kingdom). The results were interpreted according to the Clinical and Laboratory Standards Institute (CLSI) breakpoints. *Escherichia coli* ATCC 25,922 was used as a control for antimicrobial susceptibility testing.

### Multi-Locus Sequence Typing (MLST)

MLST was performed on three ESBL-producing isolates and seven non-ESBL producing isolates by amplifying the seven standard housekeeping loci *gapA*, *infB*, *mdh*, *pgi*, *phoE*, *rpoB*, and *tonB* as described previously [43]. Sequence types (STs) were assigned using the online database on the Pasteur Institute MLST website (http://bigsdb.pasteur.fr/klebsiella/klebsiella.html). The MLST profiles were analyzed and compared using BioNumerics version 7.6, created by bioMérieux (Applied Maths NV, St Martens Latem, Belgium). The minimum spanning tree predicted putative relationships among the isolates and recorded the isolates as more closely related when 6 of 7 loci were identical.

### Molecular investigations of antimicrobial resistance

The presence of ESBL genes (*bla*_CTX-M_, *bla*_TEM_, *bla*_SHV_, *bla*_GES_, *bla*_PER_, *bla*_VEB_, *bla*_OXA-1_, and *bla*_OXA-2_); MGEs genes (*IS903*, *IS26*, *intI1*, *traA*, *trbC*, *tnpU* and *tnp513*); aminoglycoside genes ((*aac)-I*, *aac(6)- I*, *aac(6')-Ib*, *ant3'-I*, *rmtA*, *rmtB*, *rmtD*, *armA*, *aadA5*, and *npmA*); quinolones genes (*qnrA*, *qnrS*, and *qnrB*); and the *bla*_CTX-M_ group surrounding regions genes (*ISEcpUP-F*, *IS26-FCJ*, *CTX-M-1-RCJ*) [40] carried by all three ESBL-producing isolates and seven non-ESBL-producing isolates were screened by PCR (Table 1). In addition, sanger methodology was performed by Sangong Biotech (Shanghai, China), and sequence analysis was performed using the National Center for Biotechnology Information (NCBI) website (https://www.ncbi.nlm.nih.gov/) with the BLAST tool.

**Table 1:** Primers used for detection and sequencing of target genes in *K. pneumoniae* isolates

Target	Forward primer (5'-3′)	Reverse primer (5′-3′)	Product size (bp)	Annealing temperature (°C)
β-Lactamases and ESBL-encoding genes				
*CTX-M*	ATGTGCAGYACCAGTAARGT	TGGGTRAARTARGTSACCAGA	593	52
*TEM*	CATTTCCGTGTCGCCCTTATTC	CGTTCATCCATAGTTGCCTGAC	800	52
*SHV*	AGCCGCTTGAGCAAATTAAAC	ATCCCGCAGATAAATCACCAC	713	52
*GES*	AGTCGGCTAGACCGGAAAG	TTTGTCCGTGCTCAGGAT	399	52
*PER*	GCTCCGATAATGAAAGCGT	TTCGGCTTGACTCGGCTGA	520	52
*VEB*	CATTTCCCGATGCAAAGCGT	CGAAGTTTCTTTGGACTCTG	648	52
MGES gene				
*IS26*	ATGAACCCATTCAAAGGCCGGCAT	TATGCAGCTTTGCTGTTACGACGG	387	55
*intI1*	CCGAGGATGCGAACCACTTC	CCGCCACTGCGCCGTTACCA	373	53
*traA*	CTGCTGTGGCGGCGTTCTTC	AGTAACCGGCGACCGACATACC	246	53
*trbC*	CGGYATWCCGSCSACRCTGCG	GCCACCTGYSBGCAGTCMCC	255	53
*tnp513*	CGAGTCAACCTCACACGCTTCC	TGCTCAATGACCTTCGGATCTTCG	269	55
*tnpU*	GCAAGGAGAAGCGACGAGTGTG	TACATGGCGGTCTCGGCTATCG	367	55
*IS903*	GCAATACGCACGCTTTCAGGC	ACTGCACGGTTACGGTCTGCA	521	55
Aminoglycoside genes				
*aac(3)-I*	ACCTACTCCCAACATCAGCC	ATATAGATCTCACTACGCGC	169	55
*aac(6’)-I*	ATGAGTGGCTAAATCGATC	CCCGCTTTCTCGTAGCA	394	55
*ant3-I*	TGATTTGCTGGTTACGGTGAC	CGCTATGTTCTCTTGCTTTTG	284	55
*aac(6')-Ib*	TGACCTTGCGATGCTCTATG	TTAGGCATCACTGCGTGTT	497	53
*rmtA*	CTAGCGTCCATCCTTTCCTC	TTTGCTTCCATGCCCTTGCC	634	55
*rmtB*	CCCAAACAGACCGTAGAGGC	CTCAAACTCGGCGGGCAAGC	565	55
*rmtD*	CGGCACGCGATTGGGAAGC	CGGAAACGATGCGACGAT	750	55
*armA*	AGGTTGTTTCCATTTCTGAG	TCTCTTCCATTCCCTTCTCC	590	55
*aadA5*	ATGGGTGAATTYTTYCCTGCACAA	TCAACGCAAGATTCTCTCATTCGT	789	55
*npmA*	CGGGATCCAAGCACTTTCATACTGACG	CGGAATTCCAATTTTGTTCTTATTAGC	334	54
Quinolone’s genes				
*qnrA*	AGAGGATTTCTCACGCCAGG	TGCCAGGCACAGATCTTGAC	578	53
*qnrS*	GCAAGTTCATTGAACAGGGT	TCTAAACCGTCGAGTTCGGCG	191	53
*qnrB*	GGMATHGAAATTCGCCACTG	TTTGCYGYYCGCCAGTCGAA	263	55
*bla*CTX-M group surrounding regions genes				
*ISEcpUP-F CTX-M-RCJ*	CAAAATGATCCCCTCGTC	AGCGGCACACTTCCTAAC	1350–2700	55
*IS26-FCJ CTX-M-RCJ*	CATTTCAAAAACTCTGCTTAC	AGCGGCACACTTCCTAAC	800–1000	55

## Data Availability

All data generated or analysed during this study were included in this published article.

## Abbreviations

AMR: Antimicrobial resistance
ATM: Aztreonam
AZM: Azithromycin
C: Chloramphenicol
CAZ: Ceftazidime
CEC: Cefaclor
CFM: Cefixime
CFP: Cefoperazone
CIP: Ciprofloxacin
CL: Cephalexin
CLSI: Clinical and Laboratory Standards Institute
CRO: Ceftriaxone
CTX: Cefotaxime
CXM: Cefuroxime
CZ: Caphazolin
*E. coli*: *Escherichia coli*
ESBL: Extended-spectrum β-lactamases
FEP: Cefepime
FOX: Cefoxitin
GTX: Gatifloxacin
HV: Hypermucoviscosity
IPM: Imipenem
K: Kanamycin
*K. pneumoniae*: *Klebsiella pneumoniae*
MEM: Meropenem
MGEs: Mobile genetic elements
MH: Minocycline
MLST: Multilocus Sequence Typing
MOX: Moxalactam
NCBI: National Center for Biotechnology Information
NOR: Norfloxacin
OFX: Ofloxacin
PCR: Polymerase chain reaction
PIP: Piperacillin
S: Streptomycin
SAM: Ampicillin/Sulbactam
STs: Sequence types
SXT: Compound Sulfamethoxazole
TE: Doxycycline
TMP: Trimethoprim
TZP: Piperacillin/Tazobactam
ZOX: Ceftizoxime
